# Direct Detection of Protein Biomarkers in Human Fluids Using Site-Specific Antibody Immobilization Strategies

**DOI:** 10.3390/s140202239

**Published:** 2014-01-29

**Authors:** Maria Soler, M.-Carmen Estevez, Mar Alvarez, Marinus A. Otte, Borja Sepulveda, Laura M. Lechuga

**Affiliations:** Nanobiosensors and Bioanalytical Applications Group, Institut Català de Nanociència i Nanotecnologia (ICN2), CSIC & CIBER-BBN, Bellaterra, Barcelona 08193, Spain; E-Mails: maria.soler@cin2.es (M.S.); mar.alvarez@cin2.es (M.A.); bert.otte@cin2.es (M.A.O.); borja.sepulveda@cin2.es (B.S.); laura.lechuga@cin2.es (L.M.L.)

**Keywords:** antibody immobilization, ProLinker™, direct immunoassay, SPR biosensor, nanoplasmonic biosensor, gold nanodisks, clinical diagnosis

## Abstract

Design of an optimal surface biofunctionalization still remains an important challenge for the application of biosensors in clinical practice and therapeutic follow-up. Optical biosensors offer real-time monitoring and highly sensitive label-free analysis, along with great potential to be transferred to portable devices. When applied in direct immunoassays, their analytical features depend strongly on the antibody immobilization strategy. A strategy for correct immobilization of antibodies based on the use of ProLinker™ has been evaluated and optimized in terms of sensitivity, selectivity, stability and reproducibility. Special effort has been focused on avoiding antibody manipulation, preventing nonspecific adsorption and obtaining a robust biosurface with regeneration capabilities. ProLinker™-based approach has demonstrated to fulfill those crucial requirements and, in combination with PEG-derivative compounds, has shown encouraging results for direct detection in biological fluids, such as pure urine or diluted serum. Furthermore, we have implemented the ProLinker™ strategy to a novel nanoplasmonic-based biosensor resulting in promising advantages for its application in clinical and biomedical diagnosis.

## 1. Introduction

During the last decades, biosensors have emerged as essential tools in biomedical applications, offering exceptional advantages over conventional clinical techniques for diagnostics and therapy monitoring. Biosensing platforms and optical biosensors in particular provide rapid, reproducible and highly sensitive detection. They allow label-free analysis (*i.e.*, photonic and plasmonic biosensors) and real-time monitoring of biological events. They have also shown exceptional potential for integration in lab-on-a-chip devices [1,2]. Among optical biosensors, surface plasmon resonance (SPR) devices have been established as routine analytical instruments due to the simplicity and versatility of the technique. Nevertheless, the increasing evolution of nanotechnology has led to novel biosensing techniques based on plasmonic nanostructured surfaces (*i.e.*, nanoplasmonics) which offer important advantages in terms of sensitivity, high throughput capabilities, integration and miniaturization [3]. So far, most research in this field has been directed towards fabrication and evaluation of the nanostructured substrates. The optimization of the biofunctionalization on the other hand is only partially addressed, limiting further development of reliable point-of-care devices with well-defined clinical applications. The challenge of an optimal biofunctionalization of the transducer is particularly relevant for immunosensors in direct configuration (*i.e.*, direct immunoassay) due to the complexity of retaining the biological activity of the antibodies (Ab) during their immobilization on a solid support [4]. The direct immunoassay should be the preferred format for diagnosis since it permits one-step analyte detection and, ideally, direct measurements from biological fluids. To fulfill this unmet need and to improve the overall efficiency of the process, many biofunctionalization strategies for antibody immobilization have been studied [4,5]. The main requirements taken into account are related to orientation control, the minimization of the chemical modification of the Ab and the reduction of the nonspecific adsorption onto the surface. Some widely used methodologies lead to random Ab orientation, decreasing the binding affinity due to blocking of the antigen recognition sites. These include physical/electrostatic adsorption, covalent linking to self-assembled monolayers or high affinity coupling between streptavidin-coated surfaces and biotinylated antibodies [4,6–8]. Direct immobilization of Fab fragments of the antibody [9] or coupling through the carbohydrate residues in the Fc part of the antibody also allow proper orientation [10]. However, both approaches require chemical modification of antibodies, which can also alter the specific binding sites and worsen the affinity. The use of Protein A or Protein G, which bind to the Fc region of antibodies [11,12], also leads to an oriented arrangement. This strategy can result in a significantly higher fraction of active antibodies since no previous modification is required. However the antibody-Protein A/G interaction can suffer instability under certain conditions. Organic molecules such as metal complexes [13] or calixarene derivatives [14] have also demonstrated their usefulness to immobilize the antibody with proper orientation avoiding chemical modification of the biomolecule. Unfortunately, in many cases these strategies are not always optimized and assessed for label-free detection in biological fluids.

In this work, we aimed to achieve a stable and robust antibody-coated surface for protein biomarker detection in biological fluids using label-free optical biosensors. We have dedicated our attention to a calixarene-based antibody immobilization strategy (ProLinker™ B). ProLinker™ B is a cup-shaped molecule first synthesized by Lee *et al.* [14], which permits binding proteins in a uniform and tight manner. Moreover, it has shown the ability to efficiently orientate and immobilize antibodies.

We have focused on assessing the ProLinker™-based strategy for plasmonic and nanoplasmonic sensor surfaces. Employing a SPR platform as a model label-free biosensor, we have carried out a preliminary comparison between different antibody immobilization strategies (*i.e.*, conventional covalent binding, Protein G-mediated capture and ProLinker™ strategy). We have evaluated the efficiency of the immobilization and the final sensitivity on direct immunoassays. The strategy has been applied for the direct detection of protein biomarkers with demonstrated relevance in clinical field, such as the human Chorionic Gonadotropin (hCG), the C-Reactive Protein (CRP) and the Focal Adhesion Kinase (FAK). A more in depth effort has been dedicated to those features barely approached so far with Prolinker™ such as the robustness and stability of the biosurface and the prevention of undesired nonspecific events in complex matrices, since the final goal is the direct detection from biological samples such as blood, serum or urine. This latter aspect has been particularly relevant in order to implement the biofunctionalization methodology on nanoplasmonic surfaces (*i.e.*, gold nanodisks on glass substrates). Besides studying the influence of the substrate on nonspecific adsorption, also comparative sensitivity studies have been performed with conventional SPR, in order to demonstrate the potential of this Localized Surface Plasmon Resonance (LSPR)-based biosensing scheme.

## 2. Experimental Section

### 2.1. Reagents

Organic solvents for chip cleaning (trichloroethylene, acetone and ethanol), sulfuric acid, hydrogen peroxide and hydrochloric acid were provided by Panreac (Barcelona, Spain). Chloroform, alkanethiols for SAM formation (16-mercaptohexadecanoic acid (MHDA) and 11-mercaptoundecanol (MUOH)), reagents for carboxylate group activation (1-ethyl-3(3-dimethylaminopropyl)carbodiimide hydrochloride (EDC) and N-hydroxysuccinimide (NHS)), ethanolamine, crosslinking molecule (bis(sulfosuccinimidyl) suberate, BS^3^), glycine, Tween 20 and bovine serum albumin (BSA) were purchased from Sigma Aldrich (Steinhem, Germany).

Human Chorionic Gonadotropin (hCG) was obtained from Abcam (Cambridge, UK), Focal Adhesion Kinase (FAK) was obtained from OriGene (Rockville, MD, USA) and C-Reactive Protein (CRP) from AntibodyBcn (Barcelona, Spain). Monoclonal antibody anti-hCG was a kind gift by the Dr. Jose Miguel Rodríguez Frade from the Department of Immunology and Oncology, National Center of Biotechnology (CNB-CSIC, Madrid, Spain). The antibody was purified by affinity chromatography using 1 mL HiTrap™ Protein G and PD-10 Desalting Columns (GE Healthcare, Uppsala, Sweden). Monoclonal antibody anti-FAK was supplied by BD Biosciences (San Diego, CA, USA) and monoclonal antibody anti-CRP was from AntibodyBcn. Recombinant Protein G was from Merck (Darmstadt, Germany). ProLinker™ B was provided by Proteogen Inc. (Seoul, Korea).

Copolymer poly-(L-lysine)-graft-PEG (PLL-PEG, MW∼75.000 g/mol) was from SuSoS (Dübendorf, Switzerland) and diamine-PEG (NH_2_-PEG-NH_2_, MW 10.000 g/mol) from Laysan Bio (Arab, AL, USA). Amine-dextran (MW 10.000 g/mol) was obtained from Invitrogen (Eugene, OR, USA). SuperBlock^®^ Blocking Buffer in TBS was purchased from Fisher Scientific (Madrid, Spain). Human serum was from Sigma-Aldrich. Urine was from healthy human donors.

Buffers used in this work are the following: PBS buffer (10 mM phosphate with 137 mM NaCl and 2.7 mM KCl, pH 7.4); acetate buffer (10 mM, pH 5.0); MES buffer (0.1 M, pH 5.4); PBST (PBS buffer + 0.5% Tween 20) and high-blocking buffer (HBB) (PBS buffer + 500 mM NaCl + 200 μg/mL BSA + 500 μg/mL amine-dextran + 0.5% Tween 20).

### 2.2. Biosensing Platforms Description

The optimization and comparison study was performed using a homemade SPR sensor platform based on the Kretschmann configuration, monitoring the binding events in real time. A polarized light of 670 nm is divided in two identical beams though a light splitter (5 mm/side cube) which are focused to the backside of the gold sensing chip (glass surface coated with 2 nm of chromium and 45 nm of gold, 10 × 10 × 0.3 mm, from Ssens, (Enschede, The Netherlands). Measurements are performed at a fixed angle of incidence and variations of the refractive index (RI) due to biointeraction events occurring at the sensing surface are detected as changes in the reflected light intensity by a multielement photodiode. The flow system consists of two flow cells (300 nL each) for independent analysis. The device incorporates all optics, electronics and fluidics components necessary to operate autonomously. Sensograms reproduce the binding event by monitoring the increase (or decrease in case of unbinding events) of the intensity of the reflected light (ΔRpp) *vs.* time. This change of the intensity of the reflected light is directly related to changes in the RI of the dielectric medium caused by mass changes on the metallic surface.

On the other hand, the nanoplasmonic biosensor is based on short-ordered arrays of gold nanodisks whose LSPR is excited in total internal reflection (θ = 70°) [15]. The arrays of gold nanodisks (D = 100 nm, H = 20 nm (Ti/Au = 1/19 nm)) were fabricated on glass substrates via hole-mask colloidal lithography (HCL) [16], assuring a LSPR wavelength (λ_LSPR_) close to 700 nm. The substrates were clamped between a trapezoidal glass prism contacting the sample through RI matching oil (*n* ≈ 1.512) and a custom-made flow cell (volume = 4 μL), which is connected to a microfluidic system consisting on a syringe pump with adjustable pumping speed that ensured a constant liquid flow and a manually operated injection valve. The biosensing surfaces were excited by a collimated halogen light source set in TE polarization for gold nanodisks and TM polarization for gold films (note that for the comparative SPR *vs*. LSPR sensitivity studies, this same setup was employed, either as an SPR sensor or as an LSPR sensor). The light reaches the substrates at a fixed angle of 70° through the prism and the reflected light is collected and fiber-coupled to a CCD spectrometer. Tracking of the real-time resonance peak position was achieved via polynomial fit using homemade readout software. The obtained sensograms show the displacement of the *λ*_(L)SPR_ to lower energy (higher RI, binding event) or higher energy (lower RI, unbinding event). The *λ*_(L)SPR_ is therefore directly related to mass changes on the gold surfaces. In both LSPR and SPR biosensors, experiments were carried out keeping a constant flow rate of 15 μL/min for immobilization and target recognition, and of 30 μL/min for regeneration. Total sample analysis was 20 min (30 min when including the regeneration of the biosurface).

### 2.3. Antibody Immobilization Strategies

Sensor chip cleaning procedure prior to biofunctionalization consisted of successive washing steps in trichloroethylene, acetone, ethanol and MilliQ water. In the case of SPR chips, the surface was then immersed in freshly prepared piranha solution (H_2_SO_4_-H_2_O_2_ 3:1) for 15 s, rinsed with water and dried under nitrogen flux. For gold nanodisks substrates the piranha step was replaced for 15 min under ozone generator.

#### 2.3.1. Covalent Strategy

Formation of a mixed alkanethiols self-assembled monolayer (SAM) was carried out ex-situ, by coating the gold chip overnight with a mixed solution of MHDA/MUOH (molar ratio of 1:20 and total alkanethiol concentration of 250 μM in EtOH). The chip was then mounted in the SPR platform in order to immobilize the antibodies by covalent binding to the carboxylic groups of the mixed SAM. Activation of carboxylate groups was performed by injecting a 0.2 M EDC/0.05 M NHS solution in MES buffer; then, antibody dissolved in PBS buffer was injected, followed by 1 M ethanolamine solution (pH 8.5) to deactivate unreacted carboxylic groups.

#### 2.3.2. Protein G Strategy

Protein G diluted in PBS (50 μg/mL) was immobilized by covalent binding to the mixed alkanethiol SAM formed as described above. Then, the antibody was injected in acetate buffer and once captured by Protein G, the crosslinker BS^3^ dissolved in PBS was flowed (1,000-fold molar excess with respect to antibody concentration). In order to quench crosslinking reaction and remove unreacted molecules, 100 mM glycine-HCl solution (pH 2.7) was injected.

#### 2.3.3. ProLinker™ Strategy

ProLinker™ layer was formed by incubating the gold chip in 3 mM ProLinker™ B solution in chloroform for 1 h at room temperature. Next, the surface was rinsed with chloroform, acetone, ethanol and water; dried with N_2_ flux and mounted on the sensor. Antibody solution in PBS buffer was injected followed by a BSA solution (100 μg/mL in PBS) to block remaining reactive free areas. For nonspecific binding studies, the concentration of the different blocking agents tested (BSA, amino-PEG, PLL-PEG and amino-dextran) was increased to 1 mg/mL. Experiments with nanoplasmonic biosensor were carried out blocking with PLL-PEG 1 mg/mL.

### 2.4. Protein Biomarker Analysis

Once immobilization process was finished, running buffer was changed to PBS and target detection was carried out. Protein samples were dissolved in PBS at different concentrations and injected in the system. For direct detection in biological samples, running buffer and dilution buffer were changed to PBST 0.5%. Regeneration of the antibody-immobilized surface took place by injecting 5 mM HCl solution.

Calibration curves were obtained measuring different concentration of target proteins by triplicate, considering the % ΔRpp as signal response in the case of SPR measurements or the Δλ_LSPR_ when using the nanoplasmonic biosensor. Curves were fitted through a non-linear one-site total binding function. Limit of detection (LOD) for each analyte was determined from the linear regression of the linear range of the calibration curve, as the concentration corresponding to the minimum measurable signal, set as three times the standard deviation of the blank signal.

## 3. Results

### 3.1. Comparison of Antibody Immobilization Strategies

**Figure 1.**
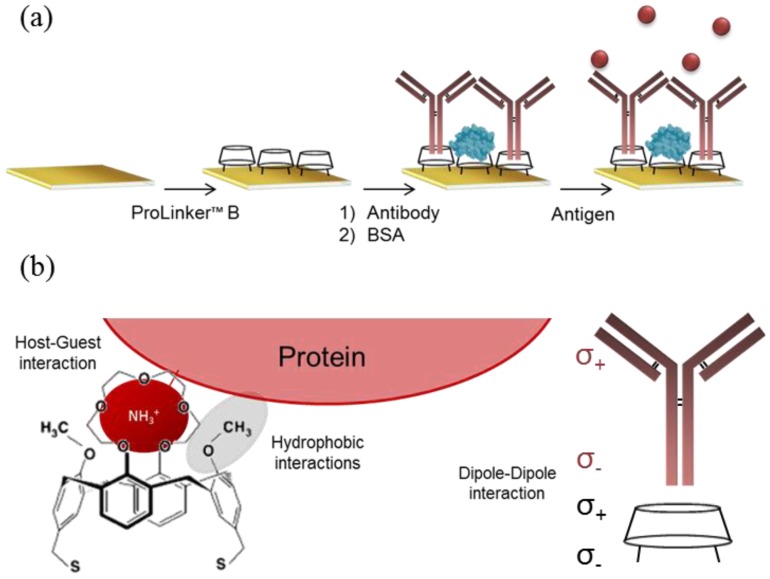
ProLinker™ strategy (**a**) Immobilization protocol: (i) surface coating with ProLinker™ B; (ii) antibody immobilization and blocking step with bovine serum albumin (BSA); and (iii) specific antigen detection; (**b**) Proposed mechanism for antibody capturing by ProLinker™ molecule. Main contribution to coupling is attributed to the host-guest interaction between ionized amino groups from the protein and the crown-ether moiety. Hydrophobic interactions between methoxy group of the linker and hydrophobic residues of the protein are also involved. End-on orientation is induced by dipole-dipole interactions. Figure adapted from [14,17].

**Figure 2.**
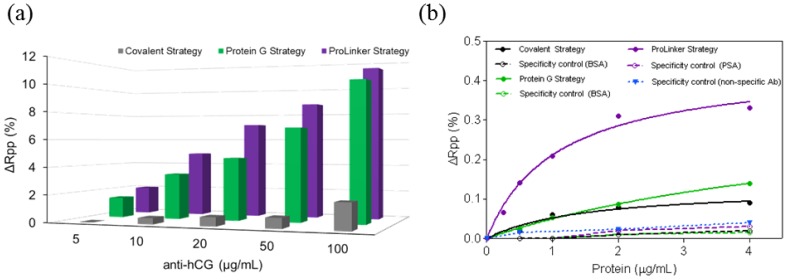
(**a**) Comparison of antibody immobilizations at different antibody concentrations (5, 10, 20, 50, 100 μg/mL) with different strategies. Grey: covalent strategy; Green: Protein G strategy ([Protein G] = 50 μg/mL); Purple: ProLinker™ strategy; (**b**) Antigen detection performed with hCG/anti-hCG for covalent strategy (black), Protein G strategy (green) and ProLinker™ strategy (purple). Concentration of anti-hCG antibody was 10 μg/mL in all cases. Dashed lines represent adsorption of nonspecific proteins onto antibody functionalized surfaces for covalent strategy (black), Protein G strategy (green) and ProLinker™ strategy (purple). Blue dotted line indicates additional control for the ProLinker™ strategy, based on the detection of hCG onto a nonspecific antibody (10 μg/mL) immobilized over ProLinker™ layer (same experimental conditions as with specific antibody).

Three different antibody-immobilization strategies were compared: Prolinker™ strategy (see Figure 1), covalent binding of the antibody, and Protein-G-based coupling. Human chorionic gonadotropin (hCG) hormone and its respective specific monoclonal antibody (mAb anti-hCG) were initially used as the model pair to carry out the study. First, different antibody concentrations, between 5 and 100 μg/mL were tested in order to compare the coupling efficiency. As can be observed in Figure 2a, Protein G and ProLinker™ strategies showed higher signal response, being the ProLinker™ 10 times higher than the covalent method using the same concentration of antibody, which indicates a significant higher antibody binding efficiency.

Sensitivity and specificity for hCG detection were evaluated and compared. An antibody concentration of 10 μg/mL was selected. The target binding with Prolinker™ strategy was considerably higher compared with Protein G and covalent approaches (See Figure 2b). The LOD achieved in each case reflected the better sensitivity of the Prolinker™ strategy (LOD = 106 ng/mL, R^2^ = 0.9647) compared with the Protein G (LOD = 427 ng/mL, R^2^ = 0.9993) and covalent strategy (LOD = 372 ng/mL, R^2^ = 0.9647).

The specificity of the antigen detection was also evaluated by performing assays with non-target control proteins (BSA and prostate specific antigen (PSA)) at different concentrations (see Figure 2b). No significant binding was observed with any of the three strategies and confirmed that signal contribution comes only from specific detection of the corresponding target. In the particular case of ProLinker™ strategy, an additional specificity test was performed, based on the immobilization of a non-specific antibody. Further injection of target protein (hCG) resulted in no signal.

**Figure 3.**
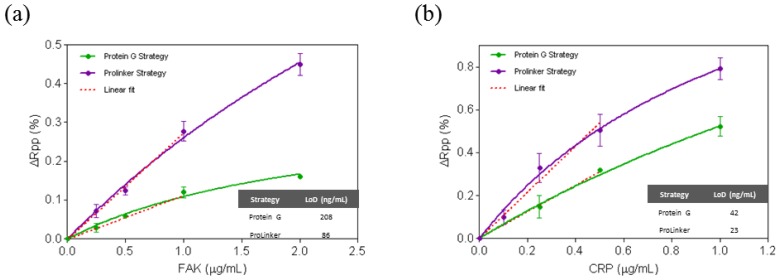
Calibration curves for (**a**) FAK protein and (**b**) CRP protein, performed with 10 μg/mL of specific antibody and following both Protein G strategy (green) and ProLinker™ strategy (purple). Dashed red lines represent linear fit of the linear region of the curves.

Immobilization strategies were further assessed for the direct detection of two protein disease biomarkers, the focal adhesion kinase (FAK) and C-Reactive Protein (CRP) and similar results were observed. Thus, under same assay conditions (*i.e.*, [Antibody] = 10 μg/mL) ProLinker™ strategy showed higher antibody immobilization signal for both antibodies (% ΔRpp(CRP) = 5.18, % ΔRpp(FAK) = 7.17), compared with Protein G approach (% ΔRpp(CRP) = 2.08, % ΔRpp(FAK) = 1.95). As can be seen in Figure 3, calibration curves for CRP and FAK biomarkers clearly showed also better sensitivities using ProLinker™, with LOD of 86 ng/mL (R^2^ = 0.9799) and 23 ng/mL (R^2^ = 0.9718) for FAK and CRP, respectively, when compared with the Protein G strategy at the same antibody concentration (LOD(FAK) = 208 ng/mL (R^2^ = 0.9895) and LOD(CRP) = 42 ng/mL (R^2^ = 0.9977)).

### 3.2. Stability and Robustness of the Direct Label-Free Immunoassay

**Figure 4.**
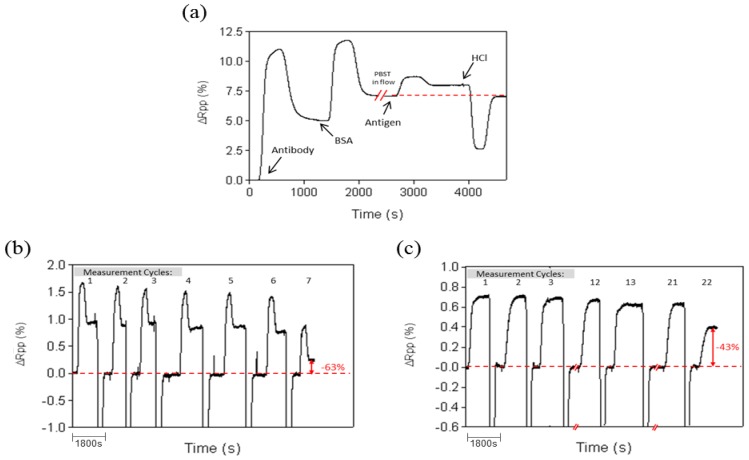
(**a**) Sensogram of biosensing measurement with ProLinker™-coated surface: antibody immobilization (anti-CRP 10 μg/mL), blocking step (BSA 1 mg/mL), antigen detection (CRP 1 μg/mL) and regeneration (HCl 5 mM); (**b**) Detection cycles performed by consecutive interaction of specific target at 1 μg/mL and regeneration with HCl 5 mM using PBS in flow; (**c**) Detection cycles performed by consecutive interaction of specific target at 1 μg/mL and regeneration with HCl 5 mM using PBST in flow.

The regeneration capability for the Prolinker™ strategy was evaluated by removing target proteins from the antibody-immobilized layer using acidic conditions (HCl 5 mM) and injecting right after the same target concentration. Experiments were performed with PBS and PBST buffer. In both cases, complete removal of target protein was observed without altering the amount of antibody on the surface (see Figure 4a), although a better stability was obtained when using PBST (up to 20 cycles) instead of PBS (up to 7 cycles).

### 3.3. Biological Samples Analysis

**Figure 5.**
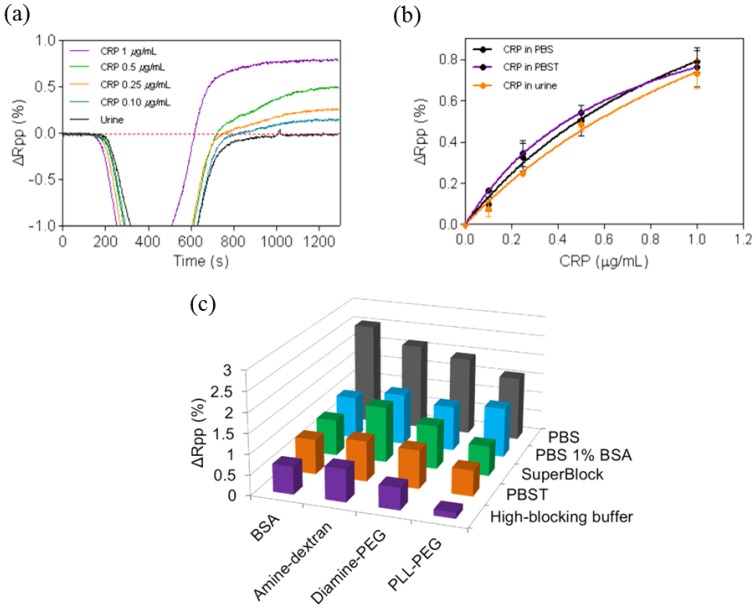
(**a**) SPR sensograms for pure urine spiked with different CRP concentrations (**b**) Calibration curves for CRP detection using ProLinker™ strategy with 10 μg/mL of specific antibody and performed in: PBS (black), PBST 0.5% (purple) and undiluted urine (orange); (**c**) Serum nonspecific adsorption onto sensor surface blocked with different agents (BSA, amine-dextran, diamine-PEG and PLL-PEG) diluted 1:10 with different buffers (PBS, PBS + 1%BSA, SuperBlock^®^, PBST 0.5% and HBB buffer).

Experiments with undiluted urine samples were done to assess the behavior of ProLinker™-based antibody layer. The experiments were done with the CRP protein. CRP immunoassays were carried out with PBST 0.5% as the running buffer. Injection of pure urine absent of protein resulted in no background (see Figure 5a), which confirms the lack of nonspecific binding onto the biofunctionalized surface. Calibration curves for CRP-spiked urine showed comparable sensitivities to the ones obtained with standard buffer conditions demonstrating that urine components did not hinder the immunochemical reaction (see Figure 5b). While the LOD in PBS and PBST was 23 ng/mL (R^2^ = 0.9718) and 22 ng/mL (R^2^ = 0.9747) respectively, a very close value was reached with pure urine (LOD = 26 ng/mL (R^2^ = 0.9973). Furthermore, regeneration with the previously selected conditions resulted in similar number of cycles (up to 20 measurements) without signal loss.

Undiluted human serum was tested over a ProLinker™-based bioactive surface (*i.e.*, Prolinker™ layer with CRP antibodies immobilized and blocked with BSA). We observed a significant increase of signal caused by nonspecific binding (data not shown). In order to minimize this effect, an optimization study was carried out, initially with diluted serum, consisting of (i) evaluating surface blocking with different antifouling compounds (BSA, amine-dextran, diamine-PEG and PLL-PEG) and (ii) changing buffer compositions used to dilute the serum. Figure 5c summarizes the results when these parameters were tested using serum at 10%. The non-specific binding could be reduced up to 94% using HBB buffer and PLL-PEG as blocking agent, in comparison with the standard conditions (PBS buffer and BSA as blocking agent).

### 3.4. Gold Nanodisks Measurements

**Figure 6.**
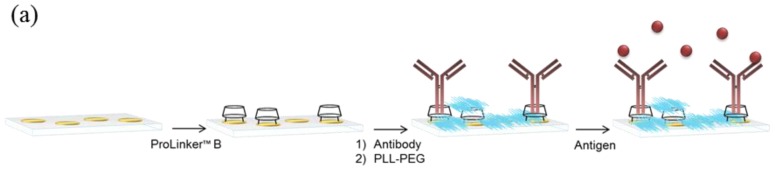

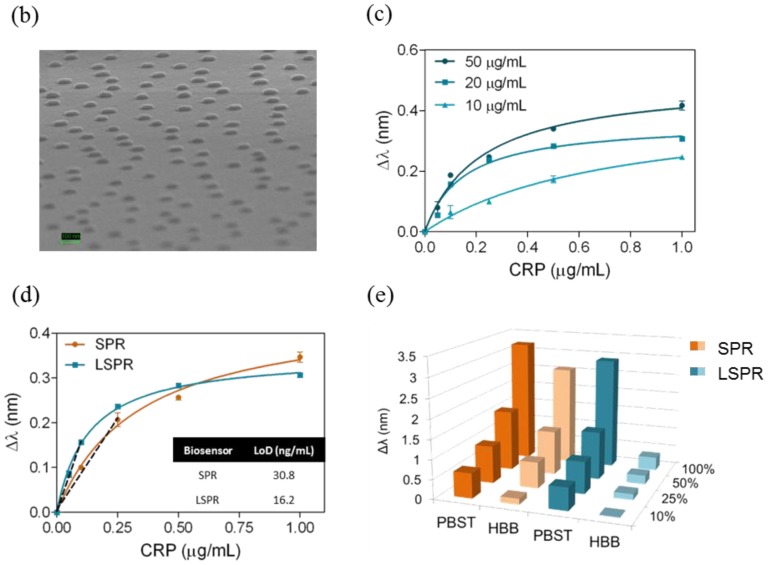
(**a**) Immobilization protocol for gold nanodisks substrates: (i) ProLinker™ layer formation; (ii) Antibody immobilization and blocking step with PLL-PEG; and (iii) Specific antigen detection; (**b**) Scanning electron microscopy (SEM) images of gold nanodisks fabricated by hole-mask colloidal lithography (**c**) CRP detection curves performed with the nanoplasmonic biosensor at different concentrations of antibody immobilized (10, 20, 50 μg/mL) with ProLinker™ strategy; (**d**) Calibration curves for CRP detection performed on gold film (orange) and gold nanodisks nanoplasmonic (blue). Antibody concentration was 20 μg/mL and PLL-PEG was employed as blocking agent for both substrates. Dashed lines represent linear fit of linear region; (**e**) Nonspecific adsorption study of serum at different concentrations (10%, 25%, 50%, 100%) using different buffers in flow (PBST 0.5% and HBB) performed for both sensor substrates: SPR gold film (orange) and LSPR gold nanodisks (blue).

The Prolinker™ strategy was evaluated on a gold nanodisks surface. We employed a homemade nanoplasmonic biosensor based on the LSPR of gold nanodisks when illuminated at a fixed angle of incidence (70°) [15]. Tracking the displacements of *λ*_LSPR_ caused by changes in the RI of the surrounding medium, enables monitoring biological events that take place on the gold nanodisks surface. By changing the sensor surface and the polarization of the light (*i.e.*, TM for gold film and TE for gold nanodisks), this biosensing device allows working in both SPR and LSPR configurations. Hence, a reliable comparison between both biosensing approaches could be performed. We have implemented the ProLinker™ strategy to functionalize nanoplasmonic substrates based on the initial results obtained with plain gold films in SPR (see Figure 6a). Several antibody concentrations were evaluated (10, 20 and 50 μg/mL). The antigen detection curves obtained with the different antibody concentrations showed increasing signals when higher amount of receptor was immobilized (Figure 6c). We selected an antibody concentration ([Antibody] = 20 μg/mL) to continue with the study. Calibration curves performed under same conditions (same antibody concentration and PLL-PEG as blocking agent) for both SPR and LSPR biosensing schemes were carried out (see Figure 6d) reaching LODs of LOD(SPR) = 30.8 ng/mL (R^2^ = 0.9859) and LOD(LSPR) = 16.2 ng/mL (R^2^ = 0.9950).

We compared the behavior of both sensor surfaces (gold film and gold nanodisks) for nonspecific adsorption of serum at different dilutions (10%–100%) when using PLL-PEG as blocking agent and those buffers that showed best results in previous experiments with SPR (*i.e.*, PBST 0.5% and HBB). Results plotted in Figure 6e showed a significant minimization of nonspecific binding onto the nanodisks substrates compared to the gold film surface when using HBB buffer in flow. A 90% reduction of the matrix adsorption was observed when injecting undiluted serum onto the biofunctionalized gold nanodisks. Similar results were observed with all the serum solutions.

## 4. Discussion

### 4.1. Assessment and Optimization of Antibody Immobilization Strategy

In biosensing, the achievement of best analytical features strongly depends on the sensor surface biofunctionalization. For label-free detection based on antibody recognition, the right orientation of the biomolecule becomes crucial. Among the myriad of antibody immobilization procedures reported, the one based on ProLinker™ molecule offers important advantages in terms of simplicity, site-specific capture and no chemical manipulation of the biomolecule. Although it has been previously compared with other strategies using fluorescence-based arrays [18], the strategy has not been optimized in-depth for label-free biosensing. This calixarene-derivative molecule forms a dense layer on gold surfaces by thiol chemisorption and proteins can subsequently specifically bind. The proposed mechanism attributes the major coupling force to a host-guest interaction between ionized amine groups of the protein and the crown-ether moiety of the linker [17]. In fact, the interaction of the ProLinker™ with several α-aminoacids has been reported [19] indicating that Ala and Val establish strong interactions due to spherical effects. Also strong and stable complexes are formed with Arg and Lys, by means of electrostatic interactions. These interactions ensure the formation of strong complexes with aminated proteins such as antibodies. In this particular case, hydrophobic interactions between the hydrophobic residues of the immunoglobulin, present in the Fc region, and methoxy groups of the ProLinker™ may also be involved in the immobilization, inducing a vertically oriented capture (see Figure 1b). A possible mechanism for the orientation of the antibodies onto the ProLinker™ layer was given by Chen *et al.* [17]. It is generally assumed that antibodies present a dipole momentum pointing from Fc to (Fab)_2_ fragment due to differences in the isoelectric point between the two regions [20]. Hence, according to the direction of the ProLinker™ dipole and antibody dipole, the immobilized antibody in an end-on orientation can interact with the ProLinker™ layer with lower energy than with other orientations. Accordingly, the sum of hydrophobic, host-guest and dipole-dipole interactions participating in antibody coupling will predictably confer both highly stable attachment and proper orientation.

Thus, in order to evaluate the efficacy of the ProLinker™ strategy (see Figure 1) to immobilize antibodies we performed a comparative test with two other conventional strategies: covalent binding to an alkanethiol SAM and affinity capture by Protein G layer. Particularly, the comparison study was focused on evaluating not only the improvement that can be achieved when appropriately orienting the antibody layer but also on evaluating the simplicity and the potential of the methodologies to generate stable and robust biofunctionalized sensor surfaces. Covalent immobilization strategy was selected as reference of a standard and commonly used procedure that generally leads to randomly oriented layer of antibodies. It inherently generates high biosurface stability and allows the control of the packing density although it usually requires high concentration of antibody, between 0.1 and 1 mg/mL [21,22]. Protein G (or Protein A)-mediated immobilization strategy has been extensively used in the biosensing field [23,24]. It shows high efficiency to appropriately orientate the antibodies although from an immobilization point of view it requires more steps, involving SAM formation, the attachment of the Protein G and subsequent binding of antibodies. Meanwhile, although the affinity is quite good [25,26], the dissociation Protein G/A–antibody occurs at extreme pH values, which usually are the conditions also required in regeneration steps to remove target from antibody. In order to generate a bioactive surface with potential for reusability, we used a crosslinker (BS^3^), which covalently binds the antibody to the Protein G molecules [27,28]. On the other hand, ProLinker™ immobilization strategy is a simple and rapid procedure that does not require any chemical modification or previous manipulation of the antibodies (see Figure 1a), which minimizes possible deterioration of the antigen affinity. Moreover, as the stability and robustness of the ProLinker™-based layer has not been so far reported we not only evaluated the final sensitivity but we completed the evaluation by assessing both reusability and reproducibility of the biosurface. We performed initial experiments with hCG hormone, a reported tumor biomarker in some cancer types such as prostate cancer, testicular cancer, breast or ovarian cancer [29,30], besides being the gold diagnostic marker for most pregnancy tests.

From the results obtained after comparing the three strategies (Figure 2), where the antibody was immobilized in a much higher extent with both ProLinker™ and Protein G strategy, we can confirm the coupling mechanism via affinity interaction leads to a more efficient binding. It is noteworthy that conventional covalent attachment resulted in very low amount of antibody on the surface (according to the low signals obtained). This is because EDC/NHS approach renders usually low yield of coupling basically due to the low density of formed NHS-esters even considering high excess of reagents. Further experiments were done with a fixed antibody concentration of 10 μg/mL, which is a relatively low concentration, considering that reported direct immunoassays usually employ concentrations around 100 μg/mL [31] or even higher [22]. hCG detection was performed and as can be seen in Figure 2b, the target binding in the ProLinker™ strategy was considerably higher compared with Protein G and covalent approaches. These results seem to indicate that ProLinker™ strategy provides an oriented antibody layer with presumably good accessibility to the active binding sites. It is worth mentioning the low detection signal observed with the Protein G-based methodology. Similar response was observed with the covalent binding, although the amount of antibody in the surface was clearly higher (about ∼11 times as can be deduced from the immobilization step). Moreover, with the Protein G the antibody should be arranged in theory in a more oriented distribution. This might be due to the crosslinking step introduced to stabilize the antibody-Protein G interaction. The crosslinking procedure consists of the formation of covalent bonds between free amine groups that are in close contact by means of BS^3^, which contains two succinimidyl groups. Amino groups of the binding sites can also suffer this cross-linking, leading to structural alterations in those areas, thereby reducing their biological activity and affinity [27]. A LOD for hCG of 106 ng/mL was reached with the ProLinker™ strategy, which would be sufficient for clinical applications (Normal concentration of hCG in men and non-pregnant women ranges from 0–5 IU/L (≈1 ng/mL)[32] and can increase to the μg/mL range [29]).

Regarding the specificity studies, control experiments were carried out using non-target proteins to assess their binding over the antibody-coated surfaces. Results summarized in the Figure 2b indicated negligible binding in all cases for the same range of concentrations than the target hCG. An additional specificity test was performed for the ProLinker™ strategy. As described before, the crown-ether moiety present in the structure couples proteins via free amine groups and, in fact, a blocking step with amino-containing molecules such as BSA is necessary to cover free ProLinker™ spaces on the surface (see Figure 1a). Therefore, in order to discard a direct binding of the target molecule onto the ProLinker™ itself, an evaluation of the cross-reactivity of the target protein to a nonspecific-previously immobilized-antibody was performed. Results depicted in Figure 2b confirmed the high specificity of the assay. As can be seen, the protein (hCG) does not bind to any component on the surface unless its specific antibody is present, showing a significant decrease in the response compared with the corresponding specific layer.

Besides hCG, two additional proteins with clinical significance as biomarkers were tested, FAK and CRP. FAK is an intracellular protein which plays an important role in cell growth and regulation. It is one of the principal molecules involved in cancer cell metastasis regulation and is associated with aggressive tumor behavior. It is known that FAK overexpression contributes to the development of typical features of malignancy in many tumors and its early detection has become a key factor in cancer diagnosis and therapy [33,34]. CRP is a widely studied marker of inflammation and infection and it is also used in heart disease risk assessment, progression and treatment effectiveness [35]. It has been also recently reported that detection of low levels of CRP in urine may be useful for the diagnosis of lower urinary tract symptoms (LUTS) [36]. Thus, detection of both biomarkers can be relevant and exemplifies two useful applications where direct detection in serum or urine is necessary. As summarized in the Results section (see Figure 3), in both cases LOD in the ng/mL were obtained with the ProLinker strategy (86 ng/mL for FAK and 23 ng/mL for CRP). Whereas no clinical ranges have been so far reported for FAK, for CRP it is well known that its concentration in human serum in healthy patients is usually lower than 10 μg/mL and it can increase to higher levels in case of inflammation, and viral or bacterial infections (10–200 μg/mL) [37]. Thus, the sensitivity achieved with this procedure is sufficient for its direct detection.

In order to complete the study, additional experiments were carried out to further assess the stability of the antibody-coated surface. It is particularly important to ensure the reusability of the sensor in case this might be necessary, either to lengthen the surface life-time, to save costs or to study the reproducibility and optimization protocols. As commented above, to our knowledge, this issue has not been addressed with ProLinker™-coated surfaces. Regeneration cycles (*i.e.*, injection of target and removal with acid conditions) were done and the corresponding signals were measured. Considering that antibody coupling to the ProLinker™ layer does not involve any covalent bond but host-guest interaction (*i.e.*, high affinity electrostatic interaction), an a priori partial loss of antibody on the surface could be expected under extreme pH conditions. Also a certain decrease of activity of the remaining bound antibody would be likely. Remarkably, we observed a complete removal of target protein without altering the amount of antibody on the surface (see Figure 4a), and retaining its activity with good levels of reproducibility. Assays with PBS as running buffer resulted in good repeatability until the seventh cycle (see Figure 4b); then a loss around 60% of the detection signal was observed and kept decreasing exponentially in subsequent measurements. It is worth mentioning that these results are considered normal in solid-based direct immunoassays, even when using covalent coupling, due to the limited stability of antibodies in aggressive mediums such as pH changes. Same experiments were then carried out with PBS buffer containing Tween 20. This additive is a surfactant widely used to reduce nonspecific binding events [38] and is usually added in conditions where detection in biological samples is the final purpose. Surprisingly, when assays were performed over ProLinker™-based surfaces with PBST (PBS with 0.5% of Tween 20) the stability of the biosurface was greatly increased, making it possible to perform up to 20 direct detection cycles with high reproducibility before reaching a 40% decrease of the signal (see Figure 4c).

From the above results, and given the high simplicity and better sensitivity obtained with ProLinker™ methodology without worsening the stability or biological activity, we continued with the assessment using biological fluids (urine and serum). We decided also to continue with CRP as protein target for this evaluation since its direct and high sensitive detection from both human fluids has important significance for several disease diagnosis and clinical relevant concentrations are already reported in the literature.

### 4.2. Direct Label-Free Immunoassay in Biological Samples

Protein biomarkers analysis is crucial in clinical diagnosis and their direct detection from biological fluids is a required demand. This analysis may become especially complex due to interferences and undesired nonspecific adsorption of matrix components present in urine, serum or whole blood. In case of label-free detection and in particular in optical biosensors, where signals are directly related with mass changes on the sensor surface, this must be minimized.

Urine is an ideal sample for disease biomarker determination as it can be obtained in large amounts with non-invasive methods. Human urine consists primarily of water with organic solutes, such as urea or creatinine, inorganic ions to a much less extent, also small organic substances and metabolites, enzymes or proteins [39]. Hence the urine matrix effect in direct analysis might be less severe than serum or plasma, where considerably higher concentrations of proteins are present. However, a high salt content like the one in urine samples may interfere in the immunochemical interaction. Potential nonspecific adsorptions cannot be discarded either [40]. Undiluted urine was initially tested. The running buffer selected for these experiments was PBST 0.5%, which provides high stability to the bioactive layer and its elevated surfactant concentration might minimize nonspecific interactions. Results in Figure 5a indicate that no components of the urine bind to the surface (no signal after addition of pure urine). Figure 5b shows calibration curves in PBS, PBST and pure urine and only slight differences were observed, leading to very similar LODs of ∼20 ng/mL. These results demonstrate that urine components did not hinder the immunochemical reaction. Overall, these positive results even with pure urine corroborate the robustness and the stability of not only the antibodies but also of the ProLinker™-based layer, resulting in an interesting candidate for the development of direct assays with views of applying in point-of-care devices.

Most protein biomarkers are not excreted in the urine but appear in blood and in those cases analysis of serum, plasma or whole blood is the best minimum invasive alternative. Direct analysis of undiluted serum (or whole blood) still remains as a challenge in direct label-free biosensing. The high amounts of proteins and lipids present in blood, which can be adsorbed onto the sensor surface, usually lead to high background signal and in the case of immunochemical interactions can also interfere in the antigen recognition. Undiluted human serum was first tested over the ProLinker™-antibody-BSA blocked layer and indeed we observed a significant nonspecific binding (data not shown). Thus we carried out an optimization study with diluted serum (10% in buffer) focused on removing the undesired adsorption. Two parameters were selected: (i) changing the blocking agent and (ii) changing the buffer composition in which the serum is diluted. The composition of the buffers is based on the blocking effect of certain compounds such as BSA or dextran, and on the presence of surfactants such as Tween 20, which reduces the nonspecific binding events. On the other hand, the blocking surface protocol was modified to include the use of antifouling compounds, such as PEGylated or dextran derivatives as blocking agents. These polymers are widely used in biomedicine because of their protein adsorption resistance and biomimetic properties [41]. In the ProLinker™ strategy, BSA is used as blocking agent to cover the remaining ProLinker™ groups with no antibody (free crown moiety of ProLinker™ will interact with amino groups present in the protein). By substituting BSA with other amine-containing compounds with more biocompatible and hydrophilic properties such as PEG/PLL-PEG and diamine-PEG) or dextran (amine-dextran), we would expect an improved behavior of the surface against serum.

As it can be seen in Figure 5c the presence of surfactants in the dilution buffer helps reducing the adsorption of serum components onto the surface (*i.e.*, PBST and HBB buffer) while the use of PLL-PEG offers a significant improvement in the antifouling resistance. A possible reason for this better behavior when compared with diamine-PEG may lie in the higher molecular weight and the relative high ratio of amine groups present in the poly-L-lysine chains of the PLL-PEG that could more efficiently cover free ProLinker™ molecules and at the same time could confer high hydrophilicity to the bioactive layer. The combination of PLL-PEG for surface blocking and HBB buffer [42], which contains blocking agents such as BSA and dextran and high salt and Tween 20 concentration, allowed a reduction of the nonspecific binding of diluted serum of 94% with respect to standard conditions (BSA as blocking agent and PBS buffer). These promising results can be considered a first approach in order to improve the conditions that permit working with more concentrated serum, or ideally with pure serum samples.

### 4.3. ProLinker Strategy Applied to Nanoplasmonic-Based Biosensor

Motivated by miniaturization and multiplexing possibilities, nanoplasmonic biosensors are considered the next-generation label-free plasmonic sensors. However, a key factor on their final development is related to the surface biofunctionalization. Nanoplasmonic sensing configurations can offer important benefits in terms of sensitivity and selectivity, but the transfer of conventional gold surface chemistry to nanostructured substrates implies additional factors, such as the substrate effects or the stronger field confinement [3], which must be taken into account when optimizing the overall performance of the biosensor.

Thus, we evaluated the behavior of the Prolinker™ strategy on gold nanodisks. We employed a homemade biosensing device which can operate both in SPR and LSPR configurations, therefore allowing a more accurate comparison between isolated nanodisks and a continuous thin gold gold layer. We followed an analogous immobilization procedure for the nanodisks (see Figure 6a), although taking into account the dual nature of the substrate (gold nanodisks on a glass layer).

We attempted a material-selective surface functionalization by exploiting the use of PLL-PEG as blocking agent. This compound has high affinity for glass surfaces [43]. Coating with PLL-PEG allowed us both the passivation of the glass surrounding the gold nanodisks and the additional blocking process of free ProLinker™ molecules (as previously observed on gold). Thus, in this way, we can generate a highly hydrophilic layer onto the sensor surface, increasing the resistance to nonspecific protein adsorption.

Gold nanodisks substrates offer a reduced active sensor surface when compared to thin gold films used in SPR biosensors (nanodisk substrates have an approximate surface occupation of 6%–7%) (see Figure 6b) [15]. Therefore, one would expect an increase of the required antibody concentration to obtain optimum antigen detection signals compared with SPR. Thus, different anti-CRP concentrations (10, 20 and 50 μg/mL) were immobilized on gold nanodisks following the same experimental procedure used in SPR device. The antigen detection curves obtained showed increasing signals when higher amount of receptor was immobilized (Figure 6c). Whereas an antibody concentration of 10 μg/mL showed similar results than 20 or even 50 μg/mL for SPR (data not shown), in the case of nanodisks, it was necessary to increase to at least 20 μg/mL to obtain better signals see Figure 6c). As seen in Figure 6d standard SPR chips showed a slightly better recognition capacity at high analyte concentration, probably due to the higher amount of antibodies immobilized on the gold film with respect to the gold nanodisks surface. However, at lower concentrations of antigen, the detection performed with the gold nanodisks provided better sensitivity, (*i.e.*, a more pronounced slope for LSPR curve than for SPR) resulting in a two-times better detectability (LOD(SPR) = 30.8 ng/mL and LOD(LSPR) = 16.2 ng/mL). This result could be partially ascribed to the strong LSPR field confinement of the nanodisks (compared to SPR), which becomes more evident at low target concentrations.

Finally, we performed a nonspecific binding study in order to evaluate the benefits of the nanostructured substrates for reducing undesired matrix adsorptions from biological fluids. We selected the serum in order to improve the obtained SPR results as much as possible. We tested different serum dilutions and serum 100%, using the two buffers with better output in SPR (PBST 0.5% and HBB). In comparison with the gold surface, a high reduction of the nonspecific adsorption was observed for the gold nanodisks in all the serum dilutions, even also in serum 100%, when using HBB buffer). Indeed, a 90% less nonspecific binding with pure serum was achieved when HBB was the running buffer. This result definitely brings out the exceptional advantages of nanoplasmonic biosensors for the direct detection of protein biomarkers. Material-selective functionalization can strongly minimize nonspecific fouling, thereby guaranteeing reliable label-free analysis in biological samples.

## 5. Conclusions/Outlook

The use of ProLinker™ as orienting molecule for antibody immobilization has been optimized for SPR biosensing and afterwards implemented on a novel nanoplasmonic biosensor showing great potential for direct immunoassay of protein biomarkers in biological fluids. This strategy turned out to be highly efficient for antibody coupling in an oriented manner with a relatively low consumption of reagent, resulting in higher analysis sensitivity in respect to more conventional methodologies. The bioactive surface generated was stable enough to allow reusability to a quite remarkable extent (up to 20 detection cycles) without the need of extra stabilization steps. The strategy offers same results regardless the antibody/target considered, proving its versatility for different targets. Moreover, ProLinker™-based layer allows direct immunoassay in undiluted urine samples avoiding nonspecific adsorption while retaining same sensitivity than in standard clean buffer conditions. These results demonstrate the robustness of the strategy even in more complex matrices and including also regeneration of the biological surface. A new approach based on the use of antifouling PEGylated compounds, particularly the copolymer PLL-PEG, instead of BSA as blocking agents, has shown encouraging results addressed to reduce undesired nonspecific binding events with serum, while retaining target detection's capability. This has been particularly remarkable with gold nanodisks surfaces. The high immobilization efficiency together with the high reproducibility and stability of the bioactive surface fulfill important requirements for label-free biosensor-based immunoassays. Overall, the implementation of the ProLinker™ strategy to gold nanodisks also highlights its exceptional potential for nanoplasmonic biosensing, additionally benefiting from the sensitivity improvements LSPR can offer.
